# Naturopathic patient care during different life stages: an international observational study of naturopathic practitioners and their patients

**DOI:** 10.1186/s12913-022-08344-0

**Published:** 2022-07-26

**Authors:** Amie Steel

**Affiliations:** Australian Research Centre in Complementary and Integrative Medicine, School of Public Health, Faculty of Health, University of Technology Sydney, Ultimo, 2006 Australia

**Keywords:** Life course perspective, Naturopathy, Preventive medicine, Healthy lifestyle, Primary health care

## Abstract

**Background:**

An individual’s health status varies with age, with most health problems increasing through different life stages. Yet, a key feature of the majority of conditions contributing burden to society globally, irrespective of life stage, is the predominance of chronic, non-communicable diseases (NCDs). An important response to this growing burden is the increasing recognition of addressing NCD prevention through a life-course perspective through primary care and public health. Naturopathy is a traditional medicine system originating from Europe, and its practitioners commonly provide primary care and focus on prevention and wellness. However, little is known about naturopathic practitioners (NPs) contribution to health care across different life stages.

**Methods:**

This secondary analysis of a cross-sectional study aimed to describe the approach to the care of NPs based on the life stage of their patients. The primary study recruited NPs from 14 regions or countries, who were invited to complete a short survey about 20 consecutive patients. The multilingual survey included the following domains: *patient demographics, reason for visit, prescribed or recommended treatments, and naturopathic interpretation of the health conditions*. Descriptive statistics were tabulated as frequencies and percentages and chi square tests were used to test associations and compare groups. Effect size was determined by Cramer’s V.

**Results:**

Participant NPs (*n* = 56) provided consultation details for 854 patients encounters. There were differences in the patient’s primary reason for visiting, the additional physiological systems the NP considered important in the management of the patient’s health, and the treatments prescribed across all life stages. However, diet (45.1–70.0%) and lifestyle (14.3–60.0%) prescription were the most common categories of treatments across all patient groups.

**Conclusion:**

NPs provide care to patients across all life stages, and diverse conditions pertinent to those life stages while also demonstrating a holistic approach that considers broader health concerns and long term treatment practices. While there may be emerging evidence supporting and informing NP clinical outcomes, the breadth and diversity of health conditions, populations and treatments within the scope of naturopathic practice underscores a need for urgent and widescale research investigating naturopathic care across the life course.

## Introduction

An individual’s health status varies with age, with most health problems increasing through different life stages [1]. For example, the burden of disease among children is more commonly attributed to conditions such as asthma and infectious disease, while adolescents experience burden from asthma but also from other conditions ranging from acne to suicide and injury [2]. Among adults, cardiometabolic and neoplastic conditions represent some of the greatest disease burdens and while these conditions continue to contribute burden in older adulthood, the elderly are also impacted by cognitive decline and musculoskeletal conditions such as osteoarthritis [2]. A key feature of the majority of conditions contributing burden, irrespective of life stage, is the predominance of chronic, non-communicable diseases (NCDs).

In response to the growing burden associated with NCDs, the focus of public health and primary care has shifted in the last 30 years to respond to the importance of an individual’s health throughout their life-course [3]. This life-course approach recognises that health develops dynamically through a process that begins before conception and continues through the lifespan [3]. The key stages of the life-course employed in this framework are preconception and prenatal care, infancy, childhood, adolescence, adulthood and older people [4]. The life-course approach originated from the study of biological programming and health inequalities using birth cohort research [5]. The cumulative outcomes of decades of life-course epidemiological investigations is now being applied to prevention and control strategies, interventions and frameworks to reduce the burden of NCDs during key life stages [4–6]. While both public health and primary care are named as central to impacting life-course health for the community, there are a number of global challenges to successfully implementing life-course perspectives in primary care, including making efficient use of the primary care workforce [7].

Ideally, primary care is characterised by integrated health services aimed at meeting people’s health needs through comprehensive care throughout the life-course and encompasses preventive, curative, rehabilitative, and palliative (including end-of-life) care [7]. However, manifesting this ideal has presented challenges to health policy makers globally, particularly in the context of integrating healthy lifestyle promotion into primary care encounters [8]. Yet, this is a challenge that primary care must overcome if it is to meaningfully address the global burden of NCDs, particularly given diet and lifestyle behaviours such as nutrition and physical activity are recognised as protective factors for individuals across most life stages with alcohol and smoking also contributing significant risk in adulthood [1, 9]. As such, the primary care workforce - all occupations engaged in the continuum of promotion, prevention, treatment, rehabilitation and palliative care [7] – must be fully utilised to achieve optimal health outcomes for the population across their life-course. A facet of the global primary care workforce that is recognised by the World Health Organisation as consistently overlooked by governments internationally is Traditional, Complementary and Integrative Medicine [10].

Naturopathy is one such traditional medicine system that originates from Europe and is practiced by more than 100,000 practitioners in over 100 countries [11]. The practice of naturopathy is defined by philosophies (e.g., holism and vitalism) and principles (e.g. *first do no harm, doctor as teacher, treat the cause, treat the person, prevention*) rather than by specific treatments [12, 13]. However, there are a number of therapies and treatments that are used more consistently than others among the global naturopathic profession, namely: (1) applied nutrition or dietary prescription, (2) lifestyle modification, (3) herbal medicine, and (4) nutritional medicine or the use of therapeutic products (e.g., tablets, powders, and liquids) of vitamins, minerals and food-based extracts for targeted clinical outcomes [14, 15]. Previous research has found individuals consult with a naturopathic practitioner (NP) for assistance with a diverse range of conditions, including illnesses contributing significant disease burden globally [15]. Research has also found NPs approach management of these conditions in a manner which aligns with their philosophies and principles by being holistic [16], patient-centred [17], and committed to patient education [18]. There is also evidence that NPs provide care to individuals at different life stages including childhood [19], pregnancy [20] and older adulthood [21]. However, this prior research has been conducted within single countries and focuses on specific life stages so does not provide a global view of the naturopathic profession within the context of life-course health. More research is needed to provide an international perspective for government about if and how they can effectively make use of NPs within the milieu of the broader primary care workforce, particularly in the context of NCD prevention and the growing recognition of a life-course approach. In response, this study presents an exploratory analysis of international data describing NPs patient profile and practice behaviours across key life stages.

## Methods

### Aim and study design

This secondary analysis of a cross-sectional study aimed to describe the approach to the care of NPs based on the life stage of their patients. Results from the primary analysis of this data have been published previously [15, 16].

### Recruitment

Naturopathic clinics in 14 regions or countries were involved in this study including those in Portugal, United Kingdom, Switzerland, Spain, Canada, United States, Chile, Brazil, Hong Kong, Australia, New Zealand, and South Africa. The World Naturopathic Federation (WNF) invited the recognised naturopathic professional associations in these countries to share an invitation pack (including an invitation email, online information sheet, online consent form, and online screening instrument) with their members. Once relevant NPs who were interested in participating in this study were confirmed that they had met the inclusion criteria for the study via the online screening instrument, an automated email was sent to the WNF. The research team then emailed the online patient-focused survey instrument link to participants.

### Survey instrument

The online survey was administered via SurveyGizmo (https://www.surveygizmo.com/). Five domains were included in this survey: *patient sociodemographics*, *reason for visit*, *interprofessional care*, *prescribed or recommended treatments*, and *naturopathic interpretation of the health condition*. The whole invitation pack and the online survey were both initially drafted in English and were translated into another three languages by native language speakers including French, Spanish, and Portuguese. The translated documents were subsequently cross-translated back to English by a second native speaker of these three languages. The research team of the World Naturopathic Federation checked the translations for accuracy with the original English documents. Surveys in different languages were all uploaded to the online platform.

### Participants

All NPs who were a member of naturopathic associations recognised by the WNF were invited to participate in this study. The inclusion criteria are (1) 5-years or more clinical practice experience; (2) preferably more than 10 patients per week; (3) having a computer terminal at the clinic. NPs were excluded if they were practising only within one specialised field or target population. The participants were asked to complete this online survey for 20 consecutive patient cases. In addition, participants were reminded, at the beginning of the second to 20th survey, whether they had missed completing a survey about the previous patient. If yes, participants were asked to provide the reason for the missed patient case.

### Survey domains

#### Patient sociodemographics

Participating NPs were asked to provide information about patients’ age groups and gender (male, female, non-binary).

#### Reason for visit

Participating NPs were asked to identify the primary presenting condition of the patient including the condition category (e.g. gastrointestinal, respiratory, and cardiovascular) and the specific condition. Based on a naturopathic clinical textbook, a total of 17 condition categories and corresponding specific conditions were provided for participants to select [22]. For each condition category, an ‘other’ option was also provided so that participants can manually enter a condition that was not included in the category list. In addition, reasons for the patient visit were included in this survey, including the visit type (initial visit, follow up) and the chronicity of the complaint (chronic, acute, unsure).

#### Interprofessional care

Participating NPs were asked, where applicable, to identify any other health professionals (general practitioner, specialist doctor, allied health professional, complementary medicine practitioner, other health professionals) known by the NP to be providing care to the patient for the presenting complaint.

#### Prescribed or recommended treatments

Based on the most common therapies reported in the Naturopathic Roots Report [23], a list of prescribed or recommended treatments to patients was also included in the survey, such as herbal medicines, dietary changes, acupuncture, and lifestyle recommendations.

#### Naturopathic interpretation of the health condition

Participating NPs were asked to indicate any other physiological systems relevant or important to the treatment of the patient’s presenting disorder. The list of physiological systems is the same as the baseline categories used to identify the reason for visit domain. Participants were able to select as many physiological system options as necessary.

### Data analysis

Survey responses were exported from SurveyGizmo so there were four separate Microsoft Excel spreadsheets (each spreadsheet for one language). All non-English responses were translated into English based on the translation developed at the beginning of the study, and all data were merged into one spreadsheet. With regards to the open text responses, all non-English answers were firstly translated using Google Translate and then cross-checked by the research team of the World Naturopathic Federation. All data were coded and imported into Stata 14.2 for analysis.

Once imported, patient age groups were recategorised to represent life stage categories: young child (0–4 years), older child (5–12 years), adolescent (13–17 years), working adult (18–65 years) and older adult (65 years or more). An additional life stage category, ‘Pregnancy and fertility’, was generated for all patients who visited the naturopath for assistance with fertility, pregnancy or postnatal health concerns. Treatment categories were collapsed into grouped variables for the following*: lifestyle and behavioural changes* (lifestyle, exercise, meditation, mind-body and rehabilitation exercise); *manual therapies* (massage, bodywork, acupuncture); *invasive treatments* (intravenous therapy, injection therapy, colonics, mesotherapy, chelation therapy, surgery); *other energetic medicines* (flower essences, tissue salts); *other traditional medicine systems* (traditional Chinese medicine, Ayurveda, humoral therapy, Unani medicine). Cumulative variables were generated for the total number of treatment categories prescribed and the total number of other health systems considered by the naturopath to be relevant or important to the patient’s primary complaint. Descriptive statistics were tabulated as frequencies and percentages and chi square tests were used to test associations and compare groups. Effect size was determined by Cramer’s V and classified according to Rea and Parker (1992) [24]: negligible association (.00 and under .10); weak association (.10 and under .20); moderate association (.20 and under .40); relatively strong association (.40 and under .60); strong association (.60 and under .80) and very strong association (.80 and under 1.00). Student’s t test analysis was used to compare the difference between life stages and both the number of physiological systems considered important in the management of the patient’s health, and the number of treatment categories prescribed.

### Ethical clearance

This project was approved by the Human Research Ethics Committee of the Endeavour College of Natural Health (#20181017).

## Results

The characteristics of participants are described in Table 1. Naturopaths from 14 countries participated. Most participants were female (62.5%), commonly aged 36–45 years (37.5%) and had been in practice between 5 and 10 years (44.6%). Participants contributed a mean of 15.1 responses.

**Table 1:** Participant characteristics (*n* = 56)

**Characteristic**	**n (%)**
Country	
*Australia*	6 (10.7)
*Brazil*	4 (7.1)
*Canada*	6 (10.7)
*Chile*	4 (7.1)
*Hong Kong*	3 (5.4)
*India*	7 (12.5)
*Nepal*	2 (3.6)
*New Zealand*	3 (5.4)
*Portugal*	4 (7.1)
*South Africa*	2 (3.6)
*Spain*	4 (7.1)
*Switzerland*	2 (3.6)
*United Kingdom*	3 (5.4)
*United States*	6 (10.7)
Gender^a^	
*Female*	35 (62.5)
*Male*	21 (37.5)
Age	
*26–35 years*	11 (19.6)
*36–45 years*	21 (37.5)
*46–55 years*	11 (19.6)
*56–65 years*	11 (19.6)
*66 years or more*	2 (3.6)
Years in clinical practice	
*5–10 years*	25 (44.6)
*11–15 years*	14 (25.0)
*16–20 years*	5 (8.9)
*21–25 years*	6 (10.7)
*26 years*	6 (10.7)
Average number of patients per week	
*Less than 10*	9 (16.1)
*11–20*	20 (35.7)
*21–30*	12 (21.4)
*31–40*	8 (14.3)
*41–50*	4 (7.1)
*51 or more*	3 (5.4)
	**Mean (SD; Min, Max)**
Number of responses per participant	15.1 (7.6; 1, 20)

^a^ No respondents identified as a non-binary person

Table 2 presents the characteristics of patients compared with their life stage. Most of the total patients were female (72.6%). This figure was slightly higher among working adults (75.0%, V = .0918, *p* = .007) but lower for other groups particularly young children (42.9%, V = .1062, *p* = .002) and children (45.0%, V = .0961, *p* = .005). Most patients in the total group were visiting the naturopath for a follow up consultation (67.0%) and this figure was higher in the elderly population (79.8%, V = .1014, *p* = .003) and lower among children (38.1%, V = .0978, *p* = .004). The majority of patients presented with a chronic health conditions (75.0%), and this rate of chronicity was even higher among elderly patients (79.8%, V = .1014, *p* = .001) but lower among adolescents (50.0%, V = .857, *p* = .045). More patients from the pregnancy and fertility (45.2%, V = .0888, *p* = .009) and elderly (36.2%, V = .0706, *p* = .039) groups were consulting with a specialist doctor compared with the total patient population (27.8%). Patients visiting a naturopath for assistance with pregnancy and fertility were more likely to be consulting with an allied health professional (26.2%, V = .0950, *p* = .005) compared to all other patients (12.4%).

**Table 2:** Characteristics of patients visiting a naturopath compared with their life stage (*n* = 854)

Characteristics	All (*n* = 854)	Young child (*n* = 21)			Older child (*n* = 21)			Adolescent (*n* = 10)			Pregnancy and fertility (*n* = 42)			Working adult (*n* = 638)			Older adult (*n* = 105)		
	N (%)	n (%)	V	*p*	n (%)	V	*p*	n (%)	V	*p*	n (%)	V	*p*	n (%)	V	*p*	n (%)	V	*p*
Sex (*n* = 851)																			
*Female*	618 (72.6)	9 (42.9)	.1062	.002	9 (45.0)	.0961	.005	5 (50.0)	.0553	.107	36 (87.8)	.0766	.025	477 (75.0)	.0918	.007	70 (66.7)	.0501	.144
*Male*	233 (27.4)	12 (57.1)			11 (55.0)			5 (50.0)			5 (12.2)			159 (25.0)			35 (33.3)		
Visit (*n* = 852)																			
*First*	281 (33.0)	9 (42.9)	−.0334	.330	13 (61.9)	.0978	.004	4 (40.0)	−.0163	.635	10 (23.8)	.0444	.195	214 (33.7)	.0262	.445	21 (20.2)	.1014	.003
*Follow-up*	571 (67.0)	12 (57.1)			8 (38.1)			6 (60.0)			32 (76.2)			421 (66.3)			83 (79.8)		
Chronicity (*n* = 844)																			
*Acute*	165 (19.6)	3 (14.3)	.0338	.617	7 (33.3)	.0555	.272	5 (50.0)	.857	.045	6 (15.8)	.0508	.337	131 (20.8)	.0572	.251	10 (9.7)	.1257	.001
*Chronic*	633 (75.0)	16 (76.2)			13 (61.9)			5 (50.0)			28 (73.7)			468 (74.2)			92 (89.3)		
*Unsure*	46 (5.5)	2 (9.5)			1 (4.8)			0 (0.0)			4 (10.5)			32 (5.1)			1 (1.0)		
Other health professionals providing care to the same patient																			
*GP (n = 854)*	369 (43.2)	10 (47.6)	.0141	.68	7 (33.3)	.0317	.355	4 (40.0)	−.0071	.837	16 (38.1)	.0235	.493	284 (44.7)	.0521	.128	47 (44.8)	.0117	.731
*Specialist doctor (n = 854)*	237 (27.8)	4 (19.1)	.0309	.367	7 (33.3)	.0198	.563	3 (30.0)	.0055	.873	16 (45.2)	.0888	.009	159 (25.0)	.1031	.003	38 (36.2)	.0706	.039
*Allied health practitioner (n = 854)*	106 (12.4)	0 (0.0)	.0598	.08	2 (9.5)	.0139	.684	2 (20.0)	.0250	.464	11 (26.2)	.0950	.005	76 (12.0)	.0229	.503	13 (12.4)	.0004	.992
*Other complementary medicine practitioner (n = 854)*	93 (10.9)	4 (19.1)	.0416	.224	1 (4.8)	.0312	.361	2 (20.0)	.0318	.352	4 (9.5)	.01	.771	66 (10.4)	.0271	.428	15 (14.3)	.0408	.233
*No other health professionals (n = 854)*	282 (33.0)	7 (33.3)	.011	.975	6 (28.6)	.015	.661	3 (30.0)	.007	.838	11 (26.2)	.033	.334	216 (34.0)	.036	.293	26 (24.8)	.0658	.055
*Any conventional health professional (n = 854)*	527 (61.4)	11 (52.4)	.0292	.393	13 (61.9)	.0018	.958	6 (60.0)	.0030	.930	31 (73.8)	.058	.089	391 (61.3)	.0023	.947	68 (64.8)	.0261	.444

The patient’s primary reason for visiting the naturopath compared with the patient’s life stage is presented in Table 3. Young children less commonly (0.0%, V = .0752, *p* = .028) presented for a musculoskeletal condition compared to the total population of patients (18.4%) while elderly patients did so more commonly (33.3%, V = .1439, *p* < .001). The frequency with which all patients presented with a gastrointestinal condition (12.1%) was less than the rate of gastrointestinal presentations in both adolescents (40.0%, V = .0928, *p* = .007) and working age patients (13.5%, V = .0715, *p* = .036). General wellness and prevention was identified as the primary reason for the patient visit in young children (23.8%, V = .1092, *p* = .001) more common than other patients (6.6%). Skin complaints were reported more frequently for patients who were young children (19.1%, V = .100, *p* = .003) and adolescents (30.0%, V = .1225, *p* < .001) compared to the total patient population (5.1%). Older children (5–12 years) were presenting with a respiratory condition (47.6%, V = .3093, *p* = <.001) significantly more frequently than all other patient categories (5.0%). Elderly patients presented with cardiovascular conditions (14.3%, V = .1880, *p* < .001) more often than the total population of patients (4.2%).

**Table 3:** Patient’s primary reason for visiting the naturopath compared with patient’s life stage, by life stage category (no analysis undertaken for Pregnancy and fertility group) (*n* = 835)

Primary Reason for visit	All (***N*** = 835)	Young child (***n*** = 21)			Older child (***n*** = 21)			Adolescent (***n*** = 10)			Working adult (***n*** = 638)			Older adults (***n*** = 105)		
	*n (%)*	*n (%)*	*V*	*p*	*n (%)*	*V*	*p*	*n (%)*	*V*	*p*	*n (%)*	*V*	*p*	*n (%)*	*V*	*p*
*Musculoskeletal condition*	158 (18.4)	0 (0.0)	.0752	.028	1 (4.8)	.0557	.103	1 (10.0)	.0235	.491	118 (18.5)	.0045	.896	35 (33.3)	.1439	<.001
*Gastrointestinal condition*	104 (12.1)	4 (19.1)	.0337	.324	2 (9.5)	.0125	.713	4 (40.0)	.0928	.007	86 (13.5)	.0715	.036	7 (6.7)	.0622	.068
*Mental health condition*	93 (10.8)	3 (14.3)	.0176	.605	5 (23.8)	.0661	.053	0 (0.0)	.0378	.268	77 (12.1)	.0679	.046	6 (5.7)	.0614	.072
*General wellness and prevention*	57 (6.6)	5 (23.8)	.1092	.001	1 (4.8)	.0119	.727	1 (10.0)	.0147	.667	45 (7.1)	.0285	.403	4 (3.8)	.0424	.214
*Female reproductive condition*	51 (5.9)	0 (0.0)	.0398	.244	0 (0.0)	.0398	.244	0 (0.0)	.0273	.424	48 (7.5)	.1141	.001	1 (1.0)	.0787	.021
*Skin/Integumentary condition*	44 (5.1)	4 (19.1)	.1000	.003	0 (0.0)	.0368	.281	3 (30.0)	.1225	<.001	34 (5.3)	.0159	.640	3 (2.9)	.0383	.261
*Respiratory condition*	43 (5.0)	2 (9.5)	.0328	.336	10 (47.6)	.3093	<.0001	0 (0.0)	.0249	.465	25 (3.9)	.1847	.013	6 (5.7)	.0121	.722
*Neurological condition*	43 (5.0)	2 (9.5)	.0328	.336	1 (4.8)	.0018	.959	1 (10.0)	.0249	.466	31 (4.9)	.0114	.737	6 (5.7)	.0121	.722
*Endocrine condition*	40 (4.7)	0 (0.0)	.035	.305	1 (4.8)	.0008	.981	0 (0.0)	.0240	.482	36 (5.6)	.0795	.020	3 (2.9)	.0319	.35
*Cancer condition*	39 (4.5)	0 (0.0)	.0345	.312	0 (0.0)	.0345	.312	0 (0.0)	.0234	.488	32 (5.0)	.0388	.255	6 (5.7j)	.021	.537
*Cardiovascular condition*	36 (4.2)	0 (0.0)	.0331	.332	0 (0.0)	.0331	.332	0 (0.0)	.0227	.506	19 (3.0)	.1028	.003	15 (14.3)	.1880	<.001
*Weight management*	34 (4.0)	0 (0.0)	.0321	.346	0 (0.0)	.0321	.346	0 (0.0)	.0220	.518	32 (5.0)	.0679	.046	0 (0.0)	.0758	.026
*Autoimmune condition*	31 (31.6)	1 (4.8)	.0098	.774	0 (0.0)	.0306	.369	0 (0.0)	.0210	.538	27 (4.2)	.0568	.096	3 (2.9)	.015	.659
*Urogenital condition*	21 (2.4)	0 (0.0)	.0251	.463	0 (0.0)	.0251	.463	0 (0.0)	.0172	.615	15 (2.4)	.0103	.763	3 (2.9)	.0100	.770
*Ageing and cognition*	10 (1.2)	0 (0.0)	.0172	.615	0 (0.0)	.0172	.615	0 (0.0)	.0118	.73	4 (0.6)	.0851	.013	5 (4.8)	.1252	<.001
*Infectious disease*	9 (0.8)	0 (0.0)	.0143	.674	0 (0.0)	.0143	.674	0 (0.0)	.0098	.773	5 (0.8)	.0059	.863	2 (1.9)	.0452	.185

The participants indicated additional physiological systems that they considered important in the management of their patient’s health (see Table 4). Compared with the frequency with which the musculoskeletal system was considered important for the total patient population (17.7%), the rate was higher in elderly patients (29.5%, V = .1162, *p* = .001). Patients who were presenting for pregnancy- or fertility-related health were more frequently identified by participants as requiring support with female reproductive (40.5%, V = .1550, *p* < .001), maternal health (33.3%, .3759, p < .001 and endocrine function (47.6%, V = .1274, p < .001) compared to all other patients (female reproductive: 15.7%, maternal health: 3.4%, endocrine: 23.8%). Weight management was identified more frequently for working adults (19.5%, V = .1044, *p* = .002) than for the full sample of patients (17.2%). The cardiovascular system (22.9%, V = .115, *p* = .001) but not female reproductive system (1.0%, V = .1517, *p* < .001) was indicated as important for elderly patients compared with the total patient population. The mean total number of systems considered important for the patients was 2.4 (SD 1.9) and this figure was significantly lower for young children (Mean 1.5, SD 1.1, *p* = .013) and children (Mean 1.3, SD 0.85, *p* = .003).

**Table 4:** Additional physiological systems considered by the naturopath to be important when managing the individual patient’s health, compared to each life stage by condition category (*n* = 854)

	**All (*n*** **= 854)**	**Young child (*n*** **= 21)**			**Older child (*n*** **= 21)**			**Adolescent (*n*** **= 10)**			**Pregnancy and fertility (*n*** **= 42)**			**Working adult (*n*** **= 638)**			**Older adult (*n*** **= 105)**		
	*N (%)*	*n (%)*	*V*	*p*	*n (%)*	*V*	*p*	*n (%)*	*V*	*p*	*n (%)*	*V*	*p*	*n (%)*	*V*	*p*	*n (%)*	*V*	*p*
*Gastrointestinal*	348 (40.8)	10 (47.6)	.0222	.517	8 (38.1)	.0086	.802	2 (20.0)	.046	.179	12 (28.6)	.0564	.100	264 (41.6)	.0286	.403	41 (39.1)	.013	.705
*General wellness and prevention*	245 (28.7)	4 (19.1)	−.0338	.323	2 (9.5)	.0673	.049	1 (10.0)	.045	.189	9 (21.4)	.0365	.286	195 (30.7)	.0761	.026	25 (25.7)	.0246	.472
*Endocrine*	203 (23.8)	2 (9.5)	.0531	.12	1 (4.8)	.0709	.038	2 (20.0)	.0096	.778	20 (47.6)	.1274	<.001	151 (23.8)	.0004	.992	20 (19.1)	.0415	.225
*Musculoskeletal*	151 (17.7)	4 (19.1)	.0057	.868	0 (0.0)	.0736	.032	1 (10.0)	.0219	.522	3 (7.1)	.0628	.066	111 (17.5)	.009	.793	31 (29.5)	.1162	.001
*Weight management*	147 (17.2)	0 (0.0)	.0724	.034	1 (4.8)	.0524	.126	1 (10.0)	.0208	.543	4 (9.5)	.0463	.176	124 (19.5)	.1044	.002	13 (12.4)	.0479	.161
*Female reproductive*	134 (15.7)	0 (0.0)	.0685	.045	0 (0.0)	.0685	.045	2 (20.0)	.0129	.706	17 (40.5)	.155	<.001	110 (17.3)	.0764	.026	1 (1.0)	.1517	<.001
*Mental illness*	132 (15.5)	4 (19.1)	.0158	.645	2 (9.5)	.0261	.446	1 (10.0)	.0164	.631	8 (19.1)	.0226	.509	95 (15.0)	.0234	.495	19 (18.1)	.0273	.424
*Cardiovascular*	108 (12.7)	0 (0.0)	.0604	.077	0 (0.0)	.0604	.077	1 (10.0)	.0087	.800	3 (7.1)	.0377	.271	78 (12.3)	.0186	.587	24 (22.9)	.115	.001
*Skin/Integumentary*	79 (9.3)	1 (4.8)	.025	.472	1 (4.8)	.0246	.472	1 (10.0)	.0028	.934	4 (9.5)	.0021	.95	63 (9.9)	.0394	.249	8 (7.6)	.0211	.538
*Autoimmune*	74 (8.7)	0 (0.0)	.0489	.153	1 (4.8)	.022	.52	0 (0.0)	.0335	.327	3 (7.1	.0213	.719	60 (9.5)	.166	.0474	7 (6.7)	.0266	.437
*Respiratory*	71 (8.3)	2 (9.5)	.007	.839	3 (14.3)	.0343	.316	0 (0.0)	.0328	.338	3 (7.1)	.0096	.778	53 (8.4)	.0020	.953	8 (7.6)	.0094	.783
*Ageing and cognition*	69 (8.1)	0 (0.0)	.0471	.169	0 (0.0)	.0471	.169	0 (0.0)	.0323	.346	1 (2.4)	.0476	.165	29 (4.6)	.2195	<.001	37 (35.2)	.3731	<.001
*Neurological*	67 (7.9)	4 (4.8)	.0182	.595	0 (0.0)	.0463	.176	3 (30.0)	.0897	.009	2 (4.8)	.0261	.446	44 (6.9)	.058	.09	14 (13.3)	.0764	.026
*Urogenital*	41 (4.8)	0 (0.0)	.0357	.297	0 (0.0)	.0357	.297	0 (0.0)	.0244	.475	3 (7.1)	.0249	.467	31 (4.9)	.0064	.851	5 (4.8)	.0007	.984
*Maternal health*	29 (3.4)	0 (0.0)	.0298	.384	0 (0.0)	.0298	.384	0 (0.0)	.0204	.551	14 (33.3)	.3759	<.001	13 (2.1)	.1268	<.001	0 (0.0)	.0702	.04
*Cancer*	29 (3.4)	0 (0.0)	.0298	.384	1 (4.8)	.012	.726	0 (0.0)	.0204	.551	0 (0.0)	.0426	.213	22 (3.5)	.0065	.85	5 (4.8)	.0282	.409
*Infectious disease*	27 (3.2)	0 (0.0)	.0287	.402	1 (4.8)	.0145	.671	1 (10.0)	.0425	.214	1 (2.4)	.0101	.767	19 (3.0)	.0165	.63	2 (1.9)	.0269	.432
	***Mean (SD)***	***Mean (SD)***		***p***	***Mean (SD)***		***p***	***Mean (SD)***		***p***	***Mean (SD)***		***p***	***Mean (SD)***		***p***	***Mean (SD)***		***p***
*Total number of systems*	2.4 (1.9)	1.5 (1.1)		.013	1.3 (0.85)		.003	1.9 (1.4)		.358	2.6 (1.5)		.391	2.4 (1.7)		.475	2.6 (1.7)		.323

The treatments prescribed to patients across life stage categories is presented in Table 5. Young children were prescribed lifestyle changes (14.3%, V = .1367, *p* < .001) or acupuncture (0.0%, V = .0970, *p* = .005) less frequently, but the rate of homeopathic medicine prescription was higher (47.6%, V = .0981, *p* = .004), than all other patients. A similar pattern of lower prescription of lifestyle changes (28.6%, V = .0909, *p* = .008) and higher of homeopathic medicines (42.9%, V = .0799, *p* = .02) was seen in the older children although there was no difference in the rate of acupuncture treatment compared with other patients. Patients presenting for assistance with pregnancy or fertility health concerns were prescribed acupuncture treatment at a significantly higher rate (47.6%, V = .1046, *p* = .002) than the rest of the patient population (27.2%). Homeopathic medicines (4.8%, V = .0947, *p* = .006) and other energetic treatments (4.8%, V = .0699, *p* = .041) were recommended much less frequently in the pregnancy and fertility group. Working adults had statistically greater incidence of being recommended lifestyle changes (60.0%, V = 1.063, *p* = .002) compared with the total population. Elderly patients were prescribed dietary changes less frequently (45.7%, V = .1136, *p* = .001) but acupuncture more frequently (41.0%, V = .1160, *p* = .001) than all other patients. The mean total number of treatment categories prescribed to the patients was 4.0 (SD 1.8) and this figure was lower in the younger children (Mean 3.1, SD 1.7, *p* = .022).

**Table 5:** Treatments prescribed or recommended by a naturopath compared to each life stage (*n* = 854)

**Treatment**	**All (*n*** **= 854)**	**Young child (*n*** **= 21)**			**Older child (*n*** **= 21)**			**Adolescent (*n*** **= 10)**			**Pregnancy and fertility (*n*** **= 42)**			**Working adult (*n*** **= 638)**			**Older adult (*n*** **= 105)**		
	*N (%)*	*n (%)*	*V*	*p*	*n (%)*	*V*	*p*	*n (%)*	*V*	*p*	*n (%)*	*V*	*p*	*n (%)*	*V*	*p*	*n (%)*	*V*	*p*
*Dietary changes*	517 (60.5)	14 (66.7)	.0199	.561	14 (66.7)	.0199	.561	7 (70.0)	.0211	.538	28 (66.7)	.0285	.405	394 (62.1)	.0526	.125	48 (45.7)	.1136	.001
*Lifestyle changes*	486 (56.9)	3 (14.3)	.1367	<.001	6 (28.6)	.0909	.008	5 (50.0)	.0152	.657	23 (54.8)	.0099	54.8	381 (60.0)	.1063	.002	56 (53.3)	.027	.430
*Nutritional medicines*	463 (54.2)	11 (52.4)	.0009	.98	13 (61.9)	.0311	.363	8 (80.0)	.0608	.076	25 (59.5)	.0338	.324	332 (52.1)	.006	.861	46 (43.8)	.0622	.069
*Herbal medicines*	445 (52.1)	9 (42.9)	.0362	.29	8 (38.1)	.0514	.133	7 (70.0)	.0345	.314	21 (50.0)	.0192	.574	347 (54.7)	.0147	.667	60 (57.1)	.0220	.52
*Acupuncture*	233 (27.2)	0 (0.0)	.0970	.005	3 (14.3)	.046	.179	1 (10.0)	.0420	.220	20 (47.6)	.1046	.002	160 (27.2)	.0754	.028	43 (41.0)	.1160	.001
*Manual therapy*	189 (22.1)	2 (9.5)	.0482	.159	3 (14.3)	.030	.381	0 (0.0)	.058	.09	9 (21.4)	.0038	.91	148 (23.3)	.0482	.159	25 (23.8)	.1050	.658
*Homeopathic medicines*	188 (22.0)	10 (47.6)	.0981	.004	9 (42.9)	.0799	.02	4 (40.0)	.0473	.167	2 (4.8)	.0947	.006	128 (20.2)	.0763	.026	26 (27.6)	.5060	.139
*Counselling and psychotherapy*	160 (18.7)	3 (14.3)	.0181	.597	3 (14.3)	.0181	.597	3 (30.0)	.0314	.358	5 (11.9)	.0398	.245	121 (19.1)	.014	.683	23 (21.9)	.0304	.374
*Other energetic treatments*	137 (16.0)	6 (28.6)	.0542	.113	3 (14.3)	.0076	.824	1 (10.0)	.0179	.600	2 (4.8)	.0699	.041	107 (16.9)	.0375	.273	13 (12.4)	.0374	.275
*Testing and investigations*	117 (13.7)	0 (0.0)	.0633	.064	6 (28.6)	.0687	.045	1 (10.0)	.0117	.732	6 (14.3)	.0039	.910	89 (14.0)	.0156	.648	14 (13.3)	.0040	.907
*Hydrotherapy*	115 (13.5)	2 (9.5)	.0183	.592	1 (4.8)	.0405	.237	2 (20.0)	.0208	.543	3 (7.1)	.0421	.218	93 (14.7)	.0588	.086	13 (12.4)	.0119	.728
*Other Traditional Medicine Systems*	110 (12.9)	0 (0.0)	.0611	.074	2 (9.5)	.0159	.642	1 (10.0)	.0094	.784	11 (26.2)	.0904	.008	74 (11.7)	.0624	.068	21 (20.0)	.0796	.02
*Invasive treatments*	58 (6.8)	0 (0.0)	.0429	.210	0 (0.0)	.0429	.210	1 (10.0)	.0139	.685	3 (7.1)	.0032	.926	38 (6.0)	.0546	.110	15 (14.3)	.1115	.001
*Other treatments*	222 (26.0)	5 (23.8)	.0079	.817	2 (9.5)	.0596	.081	3 (30.0)	.0099	.771	5 (11.9)	.0731	.033	175 (27.6)	.0607	.076	25 (23.8)	.0187	.586
	***Mean (SD)***	***Mean (SD)***		***p***	***Mean (SD)***		***p***	***Mean (SD)***		***p***	***Mean (SD)***		***p***	***Mean (SD)***		***p***	***Mean (SD)***		***p***
*Total number of treatment categories prescribed*	4.0 (1.8)	3.1 (1.7)		.022	3.5 (1.6)		.184	4.4 (2.0)		.498	3.9 (1.7)		.654	4.1 (1.8)		.159	4.1 (1.9)		.558

## Discussion

This first international study of NPs’ therapeutic approach to patients at different life stages reveals a number of interesting findings. Firstly, this analysis suggests NPs treat patients across all life stages. This finding is increasingly relevant in the context of the increased attention directed towards life course perspectives in preventive health [3, 25]. General wellness and prevention were considered by NPs when managing the health of approximately one third of patients. This was reported most commonly for patients who were working or older adults, but also frequently identified for pregnancy and fertility and young child patient groups. This attention to general wellness and prevention aligns with the life-course approach which extends from preconception through to the final years of life, and emphasises the importance of prevention and wellness in earlier life stages on an individual’s health outcomes as they age [4, 25]. However, it is important to note that the degree to which consideration of general wellness and prevention influences NPs practice decisions and prescriptions or recommendations, and the extent to which such approaches and behaviours influence a patient’s health across the life-course remains unclear. In light of this gap, and considering the acknowledged challenges of integrating prevention into primary care [7, 26] and the recent global call for such challenges to be addressed [27], further research is needed.

Diet and lifestyle changes were commonly prescribed to patients across the various life stages; two thirds of all patient groups except elderly (45.7%) were prescribed dietary changes, and lifestyle changes were recommended to at least half of patients from adolescents onwards and approximately one in three child patients but not young children. When viewed through the lens of the life-course paradigm, the inclusion of lifestyle prescription has significant potential to benefit the population, particularly if the prescriptions are tailored to respond to changing physical, social and emotional demands in a patient’s life [6]. However, there is well-established evidence that diet and lifestyle prescription is one of the most challenging therapeutic categories to use for beneficial patient outcomes [28], which may explain why it is less evident in other primary care professions [8, 29]. However, when employed effectively, diet and lifestyle prescription can produce highly impactful long term benefits for patients [30]. Certainly existing research has found patients receiving naturopathic care are more likely to make and sustain diet and lifestyle changes compared to patients receiving conventional primary care [31]. However, this previous research was focused on adult populations and as such the transferability of such outcomes to pediatric populations is not clear given the challenges associated with school, family and other infrastructure on a child’s locus of control in achieving diet and lifestyle change [32]. While parents, extended families, schools or society may have some influence over a child’s health behaviours, the power imbalances impacting the child means that overcoming the cumulative barriers to health behaviour change in pediatric populations requires coordinated effort from all sectors and as such the transferability of existing naturopathic evidence from adult populations may be limited. Overall, there were notable variations in the categories of treatments prescribed or recommended to patient groups based on their life stage, suggesting that NPs are tailoring their treatment plans to the unique needs and preferences of the patient, as has been previously reported for pediatric populations [19]. The rationale underpinning such differential treatment decisions remains underexamined and requires closer researcher attention.

The analysis found a strong association between pediatric patients - inclusive of young children, older children and adolescents – and specific health conditions such as skin and respiratory conditions. Previous research has found almost two thirds of Canadian NPs provide care to one more more pediatric patients per month, and a similar number reported receiving training in pediatric care for between one semester and one year for their clinical studies [19]. More than three quarters of Canadian NPs reported a high comfort level treating pediatric patients [19]. The reasons for pediatric patients accessing naturopathic care have not received the same research attention but may be explained by the much greater incidence of conditions such as atopic dermatitis, acne, and asthma in younger populations [33–35], coupled with patient and carer dissatisfaction with available conventional treatments for these conditions. For example, steroid treatments are commonly prescribed as a first line of treatment for inflammatory skin conditions and asthma [36, 37], and some parents hold concerns regarding the safety of steroid medications in children despite their documented effectiveness [38]. Similarly, published adverse events of other medications such as increased risk of suicide associated with the acne medication isotretinoin [39] as well as global concerns with over-prescription of antibiotics [40] may ‘push’ patients and their parents to seek other treatment options for conditions such as acne and respiratory infections [41]. In contrast, naturopathic care for these conditions embrace principles of complexity and holism [12, 13] by treating related physiological systems that may directly and indirectly impact on the presentation of a skin or respiratory condition such as the gastrointestinal system as was identified in our study and is described in naturopathic clinical texts and other practice-based research [16, 22]. Such traditional naturopathic approaches to managing these conditions are supported by emerging research which reports a clinically significant link between microbiome and allergic disease inclusive of atopic dermatitis and asthma [42]. Similarly, other naturopathic treatments have been reported to reduce symptoms in acne vulgaris, and psoriasis [43]. Likewise, there is preliminary evidence of naturopathic treatments having potential benefit in acute respiratory infections through direct immune support or symptom management [44]. Despite this evidence, there is limited research directly examining naturopathic care in the management of health conditions in pediatric populations and as such the real clinical value of such care requires investigation.

Despite the variety of categories of health conditions more commonly identified as the primary reason for working adults visiting a NP, one of the most common physiological systems or issues NPs reported considering when managing the health of their patients in this group was weight. The specific focus on weight management may be explained by the substantial international prevalence of overweight and obesity in adults [45] coupled with the recognised links between increased BMI and the onset or severity of a range of globally significant health conditions (e.g., cardiovascular disease [46], some cancers [47], mental health [48], osteoarthritis [49], etc). Despite these links, government agencies are challenged by the siloed approach to health practiced in conventional health services, driving new strategies and plans [50, 51] intended to more effectively address overweight and obesity in the future. One feature of these existing plans is a need for increased emphasis on lifestyle interventions for weight management in primary care encounters. This need may explain why lifestyle changes were recommended to a greater proportion of working adults than any other patient group in this study. While this study does not provide evidence that the various biopsychosocial factors relevant to obesity management were successfully addressed in these patients, research conducted by naturopathic researchers have explored lifestyle interventions on populations with obesity. For example, a randomised-controlled trial (RCT) from Germany investigated a 12-week yoga practice for females with abdominal obesity and found it resulted in reduced body weight, body mass index, body fat and waist-hip ratio while it increased quality of life and self-esteem compared with a wait-list control [52]. A secondary analysis of this study found the outcomes of this study was not only due to the physical activity of yoga but also due to changed dietary patterns, i.e., increased daily fruit and vegetable intake [53]. Given the global burden associated with overweight and obesity additional research investigating naturopathic treatments for this condition is needed.

In contrast to the other patient populations included in this analysis, elderly patients visiting a NP more commonly sought care for musculoskeletal conditions and were prescribed acupuncture and other invasive treatments such as prolotherapy. In addition to the breadth of evidence produced by the wider health research community with regards to non-pharmacological treatments for musculoskeletal conditions [54], naturopathic researchers have investigated a range of treatments for musculoskeletal conditions such as chronic neck pain, low back pain, fibromyalgia, and osteoarthritis among others and report a positive primary or secondary outcome in 89.3% of studies [55]. The degree to which this clinical evidence is driving patient behaviour or reflects the treatment practices used by NPs in this study, however, can not be assumed. It was also more common for NPs to consider an elderly patient’s cardiovascular health compared to other patient groups, yet the prevalence of NPs prescribing dietary changes was lowest for patients in the ‘elderly’ life stage. This practice behaviour is noted despite the strong documented association between dietary patterns and cardiovascular health [56]. While this may be explained by the primary presenting complaint being a non-cardiovascular health condition, other previous research has found that NPs considered and addressed cardiovascular conditions or risk factors if it was identified by the NP even if it was not the presenting complaint [57]. For this reason, the decreased frequency of dietary prescription in this patient population may be explained by existing evidence that elderly patients may be reluctant to change their diet due to issues associated with personal resources, psychosocial influences, and other age-related changes to their physical health [58].

### Limitations

The results of this study must be considered within the context of its limitations. While this study draws from an international sample, the diversity of naturopathic practice in specific geographical areas is likely to be impacted by cultural, social and regulatory influences. For this reason the study results should be considered within the context of these national and regional settings and may not be generalisable to the aggregate international naturopathic profession. Additional bias may also have been introduced by the self-reported nature of the survey data, as the accuracy of this data was not independently confirmed by the researchers. The target population was limited to members of professional associations that are members organisations within the WNF. This sampling frame may introduce biases in countries were regulatory mechanisms ensuring consistency in training and practice are absent. While a smaller representation of NPs from a greater number of countries was used for this exploratory study, they afforded a level of representativeness attributed to practice-based research conducted in a minimum of five locations and with at least 15 participating clinicians [59]. It should also be noted that the life stage categories used in this analysis do not reflect recommended age boundaries for life stages used in wider life-course health literature. Small sample sizes for the child and adolescent categories also limit the overall generalisability of the study findings for pediatric populations. However, as this study presents secondary analysis of data collected for other purposes it was not possible to regroup the data to more recognised life stage categories. This should be addressed through future research focussed specifically on this topic.

## Conclusions

NPs provide care to patients across all life stages, and diverse conditions pertinent to those life stages while also demonstrating a multimodal approach that may consider broader health concerns and long term treatment practices. The specific treatments employed by NPs also varies based on the patient’s life stage. While there may be emerging evidence supporting and informing NP clinical outcomes, the breadth and diversity of health conditions, populations and treatments within the scope of naturopathic practice underscores a need for urgent and widescale research investigating naturopathic care across the life course.

## Data Availability

The datasets generated and analysed during the current study are not publicly available due to intellectual property agreements but are available from the corresponding author on reasonable request.

## Abbreviations

NCDs: Non-communicable diseases
NPs: Naturopathic practitioners
WNF: World Naturopathic Federation
